# Supplementary material for: Analysis of Intracellular Magnesium and Mineral Depositions during Osteogenic Commitment of 3D Cultured Saos2 Cells
Source: Int J Mol Sci. 2020 Mar 30;21(7):2368. doi: 10.3390/ijms21072368 (PMC7177893; doi:10.3390/ijms21072368)
Supplement: Supplementary file 1 [file ijms-21-02368-s001.zip › Suppl_Info.docx]

**SUPPLEMENTARY INFORMATION**

**ANALYSIS OF INTRACELLULAR MAGNESIUM AND MINERAL DEPOSITIONS DURING OSTEOGENIC COMMITMENT OF 3D CULTURED SAOS2 CELLS**

Giovanna Picone^1§^, Concettina Cappadone^1§^, Alice Pasini^2^, Joseph Lovecchio^2^, Marilisa Cortesi^2^, Giovanna Farruggia^1,8^, Marco Lombardo^3^, Alessandra Gianoncelli^4^, Lucia Mancini^4^, Menk Ralf H^4,5,6^, Sandro Donato^4,7^, Emanuele Giordano^2#^, Emil Malucelli^1^*^#^ and Stefano Iotti^1,8#^

^1^ Department of Pharmacy and Biotechnology, University of Bologna, Bologna, Italy;

^2^	Cesena, Italy

^3^ Department of Chemistry “G. Ciamician”, Alma Mater Studiorum–Università di Bologna, via Selmi 2, Bologna, 40126, Italy

^4^ Elettra-Sincrotrone Trieste S.C.p.A., Trieste, Basovizza, Italy

^5^ INFN section of Trieste, Trieste, Italy

^6^ Department of Medical Imaging, University of Saskatchewan, Saskatoon, SK S7N 5A2, Canada

^7^ Department of physics, University of Calabria, Arcavacata di Rende (CS), Italy

^8^ National Institute of Biostructures and Biosystems (NIBB), Rome, Italy;

^§^ These authors equally contributed to this work

^#^ These authors share senior authorship

***** Correspondence: [emil.malucelli@unibo.it](mailto:stefano.iotti@unibo.it); Tel.: +39-051-209-5413

|  | % live cells | % dead cells |
| --- | --- | --- |
| Ctrl 1 day | 75.99% | 24.0% |
| Treated 1 day | 83.09% | 16.9% |
| Ctrl 3 day | 85.53% | 14.47% |
| Treated 3 day | 77.39% | 22.61% |
| Ctrl 7 day | 71.91% | 28.09% |
| Treated 7 day | 66.01% | 33.99% |
| Ctrl 14 day | 64.22% | 35.78% |
| Treated 14 day | 56.58% | 43.42% |

**Table1 SI** Percentage of cell viability of SAOS2 cells grown on board of collagen scaffold at 1,3,7 and 14 days after seeding. Cell viability was assessed by staining with eritrosin (0.1% in PBS) and performing a viability count.

| **Sample**  **name** | **Voltage**  **(kV)** | **Current (μA)** | **voxel size (μm)** | **# of projections**  **over 360°** | **Exp. time/ proj. (s)** | **Filter** | ***d_1_***  **(mm)** | ***d_2_***  **(mm)** |
| --- | --- | --- | --- | --- | --- | --- | --- | --- |
| Treated1  7 days | 40 | 200 | 5.0 | 2400 | 3.3 | None | 80 | 400 |
| Treated2  7 days | 40 | 200 | 3.3 | 2400 | 3.6 | None | 80 | 300 |
| CTRL  7 days | 40 | 200 | 5.0 | 2400 | 3.3 | None | 80 | 400 |

**Table S2: Experimental parameters use for laboratory X-ray μCT measurements.** In the table *d_1_* and *d_2_* indicate, respectively, the source-to-sample and the source-to-detector distances.

**Video S1.** 3D reconstruction of the mineral depositions in a whole control scaffold imaged by phase-contrast microfocus X-ray μCT at the TomoLab station

**Video S2.** 3D reconstruction of the mineral depositions in a whole treated scaffold imaged by phase-contrast microfocus X-ray μCT at the TomoLab station

**Video S3.** 3D reconstruction of the mineral depositions in a VOI of a scaffold imaged at the SYRMEP beamline of Elettra synchrotron light source
